# Protective effect of adipose-derived stromal cell-secretome attenuate autophagy induced by liver ischemia–reperfusion and partial hepatectomy

**DOI:** 10.1186/s13287-022-03109-2

**Published:** 2022-08-20

**Authors:** Yajun Ma, Zhihui Jiao, Xiaoning Liu, Qianzhen Zhang, Chenxi Piao, Jiayuan Xu, Hongbin Wang

**Affiliations:** 1 College of Veterinary Medicine, Northeast Agricultural University, Harbin, 150030 People’s Republic of China; 2 Heilongjiang Key Laboratory for Laboratory Animals and Comparative Medicine, Harbin, People’s Republic of China

**Keywords:** ADSC-secretome, Autophagy, IRI, Miniature pig, Laparoscopy

## Abstract

**Background:**

The therapeutic effects of adipose-derived mesenchymal stromal cells (ADSCs) may be mainly mediated by their paracrine effects. The ADSC-secretome can ameliorate hepatic ischemia–reperfusion injury (IRI). We explored the therapeutic effect of the ADSC-secretome from the perspective of excessive hepatocyte autophagy induced by hepatic IRI.

**Methods:**

We established a miniature pig model of hepatic ischemia–reperfusion (I/R) and hepatectomy using a laparoscopic technique and transplanted ADSCs and the ADSC-secretome into the liver parenchyma immediately after surgery. Liver injury and hepatocyte autophagy were evaluated by histopathological examination and assessment of relevant cytokines and other factors.

**Results:**

The results showed that the ADSC-secretome alleviated the pathological changes of liver tissue and the microstructural damage of hepatocytes after IRI. Moreover, the expression levels of autophagy-related markers including Beclin-1, ATG5, ATG12, and LC3II/LC3I decreased, whereas those of p62 increased during phagophore expansion. Furthermore, the expression levels of markers related to the autophagy inhibition pathway phosphatidylinositol-3-kinase/Akt/mammalian target of rapamycin (PI3K/Akt/mTOR), including PI3K, Akt, and mTOR, increased.

**Conclusion:**

The ADSC-secretome attenuates hepatic I/R and hepatectomy-induced liver damage by inhibiting autophagy, which is possibly mediated by activation of the PI3K/Akt/mTOR signaling pathway. In addition, there was no significant difference between ADSCs and the ADSC-secretome in the regulation of hepatocyte autophagy. Therefore, ADSCs may improve the excessive autophagy-induced injury of hepatocytes in hepatic I/R and hepatectomy through paracrine effect. Our findings provide new insight into the therapeutic potential of cell-free products, which could replace cell therapy in liver diseases.

**Supplementary Information:**

The online version contains supplementary material available at 10.1186/s13287-022-03109-2.

## Introduction

Liver ischemia–reperfusion injury (IRI) is a serious complication that exerts a negative impact on the prognosis of patients during hepatectomy and liver transplantation [1]. In the early stage of liver IRI, ischemia-induced deprivation of nutrition and oxygen causes hepatocyte damage. With the decrease in ATP production, cell metabolism slows down, anaerobic glycolysis is activated, and intracellular enzymes, such as phospholipase C and protein kinase C, are activated, inducing liver necrosis and apoptosis. The pH imbalance and accumulation of ions caused by the dysfunction of ion channels lead to a change in mitochondrial permeability. After reperfusion, neutrophils and macrophages are activated and accumulate in the liver. These cells increase IRI by secreting paracrine and autocrine signals, such as reactive oxygen species and inflammatory cytokines [2, 3]. The systemic effects of IRI, as well as those on the liver, are of great significance in the clinical application of liver transplantation [4].

Autophagy is a highly conserved biological process in eukaryotic cells that maintains cell homeostasis and viability by recycling and reusing energy [5, 6]. It is usually considered a cell protection mechanism, however, excessive autophagy will lead to autophagic cell death [7, 8]. Studies have shown that autophagy can either promote cell survival or death according to the cell type, environmental conditions, and specific stimuli [9]. The liver is largely dependent on pathological and physiological autophagy. Therefore, autophagy is of great significance in the pathogenesis of liver diseases and normal liver physiological processes. Furthermore, these findings provide a potential new strategy for improving the effects of IRI, which can be achieved by regulating the level of autophagy [10, 11]. The phosphatidylinositol-3-kinase/Akt/mammalian target of rapamycin (PI3K/Akt/mTOR) pathway is one of the key signaling pathways regulating autophagy. Growth factors inhibit autophagy by stimulating the PI3K/Akt pathway and activating mTOR.

With the development of stromal cell biology and technology, regenerative medicine seeks to stimulate the wound-healing response to injury to restore the normal tissue structure and function. Many tissue damage model studies have shown that exogenous injection of adipose-derived mesenchymal stromal cells (ADSCs) into injured tissues can promote tissue regeneration, angiogenesis, and functional recovery [12, 13]. It has been demonstrated that the therapeutic potential of ADSCs transplantation into host tissues may be primarily a result of their secretome effects rather than that of cell replacement and differentiation alone [14, 15]. Furthermore, the ADSC-secretome can stimulate cell proliferation, inhibit apoptosis, promote angiogenesis, and inhibit inflammation and the immune response [16]_._ The ADSC-secretome promoted liver injury repair through multiple mechanisms, such as anti-fibrotic and anti-inflammatory processes, and by promoting liver regeneration [17, 18]. Recently, ADSCs have been shown to ameliorate liver tissue injury by reducing excessive autophagy in hepatocytes in a model of hepatic I/R and hepatectomy [19]. To further explore the paracrine role of ADSCs in the treatment of excessive autophagic injury caused by hepatic I/R and hepatectomy, we established a model of hepatic I/R and hepatectomy in miniature pigs and transplanted the ADSC-secretome as an intervention into the liver tissue of animals. The ADSC-secretome attenuated liver pathology and cell injury by inhibiting excessive autophagy-induced injury. Our findings provide new insight into the therapeutic potential of cell-free products, which could replace cell transplantation, in liver diseases.

## Materials and methods

### Animals

Twenty-four Bama miniature pigs aged 4–6 months, weighing 20–30 kg, half male and half female, were provided by the Bama Miniature Pig Farm of the College of Life Sciences (Harbin, China). All animals were raised under the same conditions and randomly divided into the IRI (IRI), DMEM control (DMEM), ADSC-secretome treatment (ADSC-sec), and ADSC treatment (ADSCs) groups (six animals per group). The animals were raised in the same controlled environment with ambient temperature and humidity, 12 h light/dark cycle, and free access to piglet food and water. The study received ethical approval and was carried out in accordance with the Helsinki Declaration. All experiments were approved by the Animal Care and Use Committee of the Northeast Agricultural University (approved by the State Council on October 31, 1988, and promulgated by Decree No. 2 of the State Science and Technology Commission on November 14, 1988). All methods were performed in accordance with the approved guidelines.

### ADSC culture and preparation of the ADSC-secretome

The adipose tissue of Bama miniature pigs was obtained under sterile conditions and washed with PBS solution. The fascia and blood vessels were removed, and the tissue was cut into pieces and digested with 0.01% collagenase type I. Then, it was resuspended in L-DMEM supplemented with 10% FBS (Clark, USA), 100 µg/ml streptomycin, 1 µg/ml penicillin, and 2 mM L-glutamine (Solarbio, China). Finally, the cells were cultured in an incubator at 37℃ with 5% CO2. The ADSCs were identified on the basis of morphology, immunophenotype, and differentiation potential [20]. In this study, adipose tissue was obtained from 5 Bama minipigs and cultured ADSCs. According to different pig donors, the primary cultured ADSCs were divided into 5 batches (N1-N5), and the total protein concentration of ADSC-secretome was tested (See Additional file 1: Table A). In order to better compare the therapeutic effect between ADSC-secretome and ADSCs, each batch of ADSCs cultured to the fourth passage was divided into 2 parts. One part of ADSCs (1 × 10^6^ cells/kg) was used as the ADSCs group intervention. The other part of ADSCs (1 × 10^6^ cells/kg) was placed under serum-free starvation conditions. After starvation for 48 h, the medium was harvested, centrifuged, and filtered for purification to remove dead cells and cell debris. Finally, the purified medium was extracted and put into a 3KD concentrating tube (Millipore, Billerica, USA), centrifuged (5000 g, 1.5 h, 4 °C) for concentration, and the concentrated supernatant was ADSC-secretome (ADSC-sec group intervention). The liquid volume ratio before and after concentration is 24:1. The N3 batch of ADSCs (twice as many cells as other batches) was divided into 4 parts for the intervention of 2 pigs each in the ADSCs group and the ADSC-sec group.

### Surgical procedure

The miniature pigs were kept on the operating table in a supine position, and the temperature of the operating table was maintained at 37 ℃. Intraoperative anesthesia was used, and then, laparoscopic surgery, a minimally invasive surgical technique, was used for model construction, which involved the induction of right hemi-hepatic ischemia for 60 min, and left hemi-hepatectomy [21]. Our previous in vivo tracing result of ADSCs found that 24 h after transplantation, ADSCs were observed in different parts of the right hepatic lobe. Consistent with previous studies [22], this study used liver parenchyma multipoint injection. The animals in the IRI, DMEM, ADSCs, and ADSC-sec groups received, respectively, saline, DMEM, ADSCs, or ADSC-secretome multipoint injections into the liver parenchyma using laparoscopic instruments immediately after the construction of the hepatic I/R combined hepatectomy model. ADSCs of P3-P5 were resuspended in saline for allotransplantation in the ADSCs group. As DMEM was the basic medium used to culture cells, the control group for the ADSC-sec group was injected with concentrated DMEM medium. The tissue samples were collected from the same location in the right lobe of the liver preoperatively (after anesthesia) and one day after the operation under the same environmental conditions and stored at -80 ℃. All operations were performed using the laparoscopic technique.

### Histological analysis

The collected liver tissue samples were fixed in 4% paraformaldehyde for 24 h, paraffin embedded, sectioned, and stained with hematoxylin and eosin (H&E). Finally, the morphological changes of the liver tissues in each experimental group were observed under light microscopy, focusing on whether or not the structure of hepatic lobules was intact, hepatocytes showed degeneration and necrosis, and inflammatory cell infiltration and cytoplasmic vacuolization were present.

### Transmission electron microscopy (TEM)

The liver tissue samples were trimmed into 1 mm^3^ tissue blocks, double fixed with 2.5% glutaraldehyde and 1% osmic acid, dehydrated at 4 ℃, soaked at room temperature, paraffin embedded, sectioned (thickness 50–60 nm), placed on 200 mesh copper mesh, and double stained with uranium acetate–lead citrate. Finally, the sections were observed using an H-7650 electron microscope (Hitachi, Japan) and images were obtained.

### Western blotting

The protein lysate was prepared by adding PMSF (Beyotime, Shanghai, China) and phosphatase inhibitor (MCE, Monmouth Junction, USA) to the tissue protein extraction reagent at a volume ratio of 100:1. The liver tissue samples were placed into the prepared protein lysate, homogenized using a tissue homogenizer, and lysed at 4 ℃ for 30 min. The supernatant was centrifuged to obtain the total protein of the samples. The total protein concentration of each experimental group was measured using the bicinchoninic acid (BCA) protein quantitative method (Beyotime, China). Sodium dodecyl sulfate–polyacrylamide gel electrophoresis (SDS-PAGE) was prepared for the electrophoretic separation of protein molecules with different molecular weights, and then, nitrocellulose (NC) membranes (Biosharp, China) were used to transfer the target proteins. The membranes were placed and blocked in 5% skim milk solution for 2 h at room temperature and then washed with TBST and incubated with the following primary antibodies: β-actin (Cell Signaling Technology, USA), Beclin-1, p62, ATG5 (Wanleibio, China), LC3 (Novus Biologicals, USA), Akt, p-Akt, mTOR, and p-mTOR (ABclonal Technology, USA) overnight at 4 ℃. Then, the membranes were washed with TBST and incubated with the appropriate horseradish peroxidase (HRP)-conjugated secondary antibody (1:7500, ImmunoWay, USA) for 2 h. Finally, the membranes were incubated with the Western Bright ECL reagent (Advansta, USA) and imaged using a Tanon 5200 Imaging System (Tanon Science and Technology Co., Ltd., China). In this experiment, β-actin was used as an internal reference. The image processing program ImageJ was used to analyze the gray level of strips.

### Immunohistochemistry

The expression level of LC3 in liver tissue was detected using immunohistochemistry. Liver tissue samples were cut into appropriate sizes, fixed in 4% paraformaldehyde for 24 h, and then paraffin embedded and sectioned. The sections were dewaxed in an 80 ℃ oven overnight, and immersed in 3% H2O2 solution for 10 min in the dark for endogenous peroxidase blockade, followed by antigen repair in a pressure cooker using sodium citrate antigen repair solution. Sections were sealed for 20 min at room temperature with BSA, incubated with primary antibody (1:2000, LC3, Novus Biologicals, USA) overnight at 4 ℃, followed by incubation with streptavidin-labeled HRP for 30 min at room temperature. Finally, the sections were stained with DAB and hematoxylin, sealed with neutral glue, and dried in an oven. The stained sections of each group were observed under a microscope and analyzed using the Image-Pro Plus 6.0 software (Media Cybernetics, USA).

### Real-time quantitative PCR

Total RNA was extracted from the liver samples using the TRIzol reagent (Invitrogen, China). The quality and concentration of the RNA were assessed using NanoDrop™ One/One (Thermo Fisher Scientific, USA). The total RNA was reverse transcribed into cDNA using the ReverTra Ace qPCR RT Master Mix (Toyobo, Japan). Then, using the cDNA as the template, RT-qPCR was carried out in a LightCycler 480 (Roche, Germany) according to the manufacturer's instructions to detect hepatocyte-specific genes in the liver tissue samples. The reaction program was as follows: 15 s at 95 °C for pre-denaturation, 40 cycles of 5 s at 95 °C for denaturation, and 60 s at 60 min for annealing and elongation. The primers were synthesized by Sangon Biotech (Shanghai, China) and are listed in (Table 1).

**Table 1:** Gene-specific primers used in the real-time quantitative PCR

Gene	Primer sequences (5′-3′)
Beclin-1	Forward 5′-TCATGCGATGGTGGCTTTCC-3^′^ Reverse 5′-ATGGAATAGGAGCCGCCACT-3′
ATG5	Forward 5′-ACCTTTGCAGTGGCTGAGTG-3′ Reverse 5′-TCAATCTGTTGGTTGCGGGA-3′
ATG12	Forward 5′-CAACTGCTGCTGAGGGCGATG-3′ Reverse 5′-CACCGGCAGGTTCTTCTGTTCC-3′
P62	Forward 5′-CTGATGAAGGTGGCTGGCTGAC-3′Reverse 5′-CAAGGGCGGTGGGTGTTTCG-3′
PI3K	Forward 5′-ACGGAGGAGGTGCTCTGGAAC-3′ Reverse 5′-GGACTCGGGACTGGGCATCTC-3′
Akt	Forward 5′-GACGGCACCTTCATCGGCTAC-3′ Reverse 5′-CGCCACGGAGAAGTTGTTGAGG-3′
mTOR	Forward 5′-GCACGTCAGCACCATCAACCTC-3′ Reverse 5′-GCCTCAGCCATTCCAACCAGTC-3′

### Statistical analysis

All the data were analyzed using GraphPad Prism 7.0 (GraphPad Software, USA). All values are expressed as the mean ± SD (standard deviation). Comparisons between groups were assessed using ANOVA (one-way analysis of variance). A *P* value of < 0.05 was considered statistically significant.

## Results

### The ADSCs/ADSC-secretome protects pigs from hepatic I/R and hepatectomy injury

**Fig. 1:**
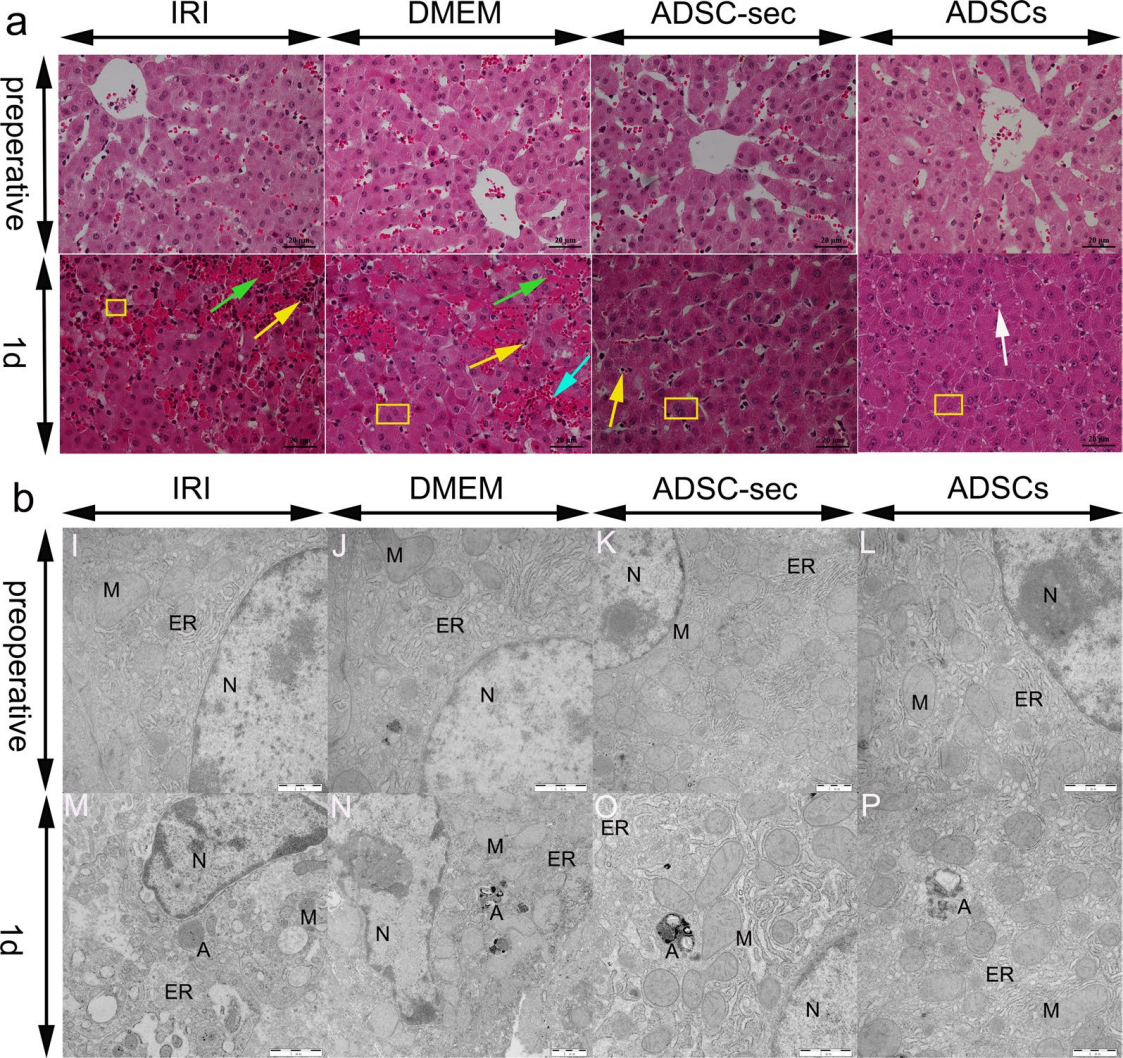
Histopathological changes and ultrastructural changes in the liver post-IRI. **a**: HE-stained liver tissues. Green arrows indicate necrosis, the white arrow indicates hepatocyte vacuolar degeneration, the blue arrow indicates hemorrhage, yellow rectangle indicates hepatocyte swelling, and yellow arrows indicate inflammatory cell infiltration (Magnification × 400). **b**: Transmission electron microscopy micrographs of the liver. A: Autophagy structure; N: nucleus; ER: endoplasmic reticulum; and M: Mitochondria (magnification: 12,000 ×). IRI: ischemia–reperfusion injury; ADSC-sec: adipose-derived mesenchymal stromal cell-secretome; and ADSCs: adipose-derived mesenchymal stromal cells

As shown in Fig. 1a, the preoperative histological evaluation revealed that the structure of hepatocytes was intact and the morphology of hepatocytes was normal. On postoperative day one, severe liver tissue damage was caused by hepatic I/R and hepatectomy. However, a significant improvement was observed in the ADSCs and ADSC-sec groups. The results of postoperative day one TEM observation showed that different degrees of hepatocyte damage were observed in each group. In addition, obvious autophagic structures were observed in all groups. As shown in Fig. 1b, the hepatocytes of the IRI and DMEM groups showed abnormal morphology with nucleus shrinkage and deformation, swelling of the endoplasmic reticulum and mitochondria, even disappearance of the mitochondrial cristae structure. The overall hepatocyte structure of the ADSCs and ADSC-sec groups was relatively intact, the damage was mild, the swelling of the mitochondria and endoplasmic reticulum was not serious, and the nuclear structure was relatively normal.

### The ADSCs/ADSC-secretome inhibits autophagy in Hepatic I/R and hepatectomy injury

**Fig. 2:**
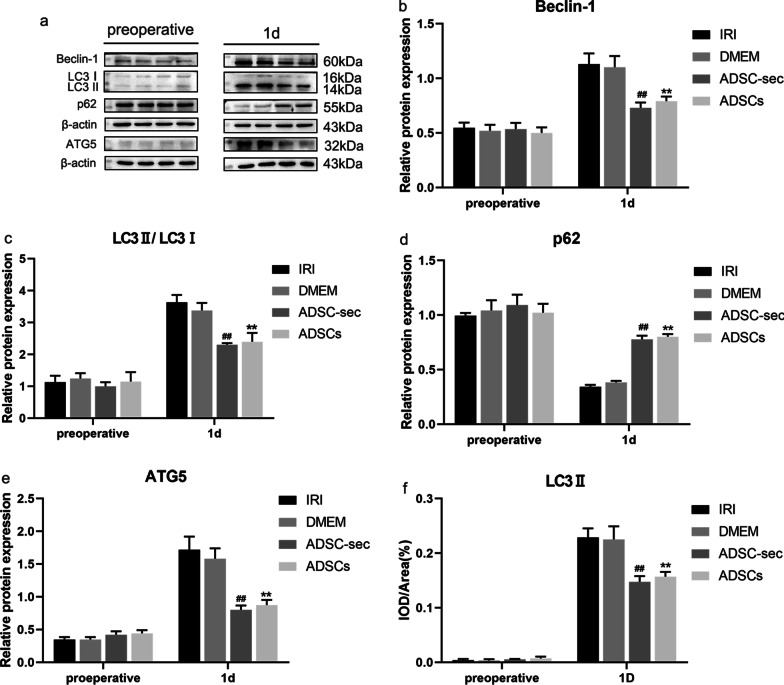
Effect of ADSC-secretome transplantation on autophagy-related protein levels. **a**: Representative western blot analysis of Beclin-1, LC3II/LC3I, p62, and ATG5. **b**–**e**: Quantification of Beclin-1, LC3II, P62, and ATG5. **f**: Analysis of LC3II protein immunohistochemical results. **P < 0.01, vs. IRI group. ^##^*P* < 0.01, vs. DMEM group. IRI: ischemia–reperfusion injury; ADSC-sec: adipose-derived mesenchymal stromal cell-secretome; and ADSCs: adipose-derived mesenchymal stromal cells

As shown in Fig. 2, hepatic I/R and hepatectomy injury enhanced autophagy on postoperative day one. The protein expression levels of Beclin-1, LC3II/LC3I, ATG5, and p62 in liver homogenates in the DMEM group on postoperative day one were not significantly different from that in the IRI group (*P* > 0.05). As expected, Beclin-1, LC3II/LC3I, and ATG5 levels were significantly decreased (*P* < 0.01) and the level of p62 was significantly increased (*P* < 0.01) on postoperative day one in ADSCs-treated pigs when compared with those of the IRI groups. And Beclin-1, LC3II/LC3I, and ATG5 levels were significantly decreased (*P* < 0.01), and the level of p62 was significantly increased (*P* < 0.01) on postoperative day one in ADSC-secretome-treated pigs when compared with those of the DMEM groups. In addition, the protein expression analysis results showed that there was no significant difference in the expression of autophagy-related protein factors Beclin-1, LC3II/LC3I, ATG5, and p62 between the ADSCs and ADSC-sec groups on postoperative day one (*P* > 0.05). Furthermore, as shown in Fig. 2f, the results of immunohistochemistry are consistent with the results of western blotting. For the images of immunohistochemistry, see Additional file 1: Fig. A.

**Fig. 3:**
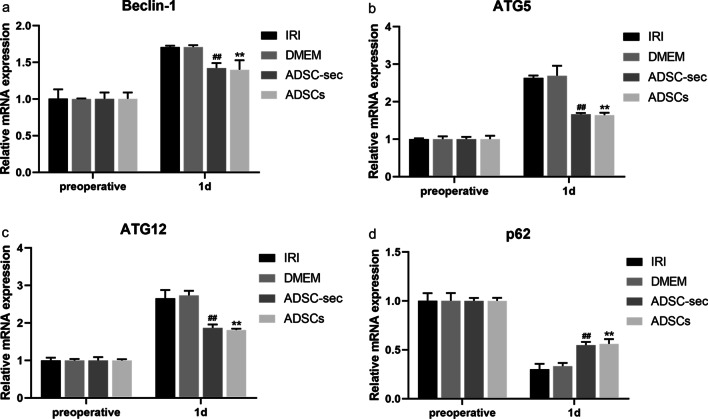
Effect of ADSC-secretome transplantation on autophagy-related mRNA levels. **a**–**d**: RT-qPCR results showing Beclin-1, ATG5, ATG12, and p62 mRNA levels. **P < 0.01, vs. IRI group. ^##^*P* < 0.01, vs. DMEM group. IRI: ischemia–reperfusion injury; ADSC-sec: adipose-derived mesenchymal stromal cell-secretome; and ADSCs: adipose-derived mesenchymal stromal cells

We further investigated autophagy by analyzing autophagy-related gene expression using reverse-transcription quantitative PCR (RT-qPCR). As shown in Fig. 3, no significant difference was observed in the levels of all detected factors between the DMEM and IRI groups (*P* > 0.05). Consistent with the autophagy-related protein expression results, the levels of the autophagy-related genes Beclin-1, ATG5, and ATG12 were significantly downregulated and p62 was significantly upregulated in the ADSCs/ADSC-sec groups on postoperative day one when compared with those of the IRI/DMEM groups (*P* < 0.01). Similarly, there was no significant difference in these gene levels between the ADSCs and ADSC-sec groups on postoperative day one (*P* > 0.05).

### The ADSCs/ADSC-secretome activates PI3K/Akt/mTOR signaling in hepatic I/R and hepatectomy injury

**Fig. 4:**
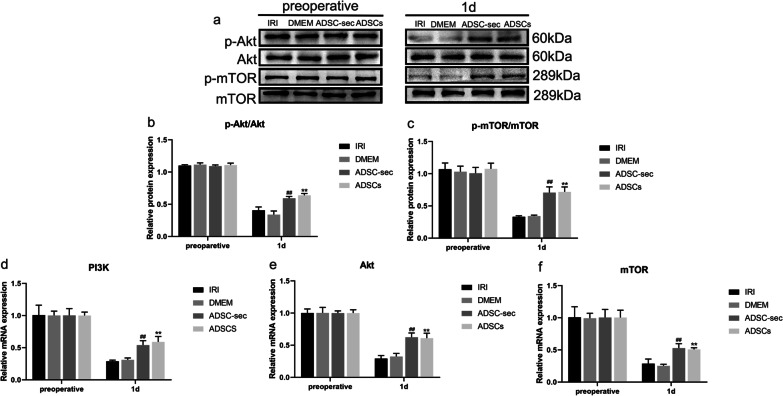
Effect of ADSC-secretome transplantation on autophagy-related pathway PI3K/Akt/mTOR protein and mRNA levels. **a**: Representative western blot analysis of p-Akt, Akt, p-mTOR, and mTOR. **b**–**c**: Quantification of p-Akt/Akt and p-mTOR/mTOR. **e**–**f**: RT-qPCR results showing PI3K, Akt, and mTOR mRNA levels. **P < 0.01, vs. IRI group. ^##^*P* < 0.01, vs. DMEM group. IRI: ischemia–reperfusion injury; ADSC-sec: adipose-derived mesenchymal stromal cell-secretome; and ADSCs: adipose-derived mesenchymal stromal cells

Western blot and RT-qPCR (Fig. 4) analyses identified that the PI3K/Akt/mTOR pathway was repressed on postoperative day one under hepatic I/R and hepatectomy injury. The results showed no significant changes in p-Akt/Akt and p-mTOR/mTOR protein expression and mRNA expression of PI3K, Akt, and mTOR in the DMEM group on postoperative day one compared with those of the IRI group (*P* > 0.05). In the ADSCs/ADSC-sec groups, the ADSCs/ADSC-secretome sharply elevated the levels of p-Akt/Akt and p-mTOR/mTOR and the gene expression of PI3K, Akt, and mTOR compared with those of the IRI/DMEM groups on postoperative day one (*P* < 0.01). According to these results, there was no significant difference in the levels of p-Akt/Akt and p-mTOR/mTOR between the ADSC-sec and ADSCs groups on postoperative day one (*P* > 0.05). Moreover, the PI3K, Akt, and mTOR mRNA levels were examined after ADSCs and ADSC-secretome treatment using RT-qPCR and the results showed that the PI3K, Akt, and mTOR mRNA expression levels were not significantly different between the two treatment groups on postoperative day one (*P* > 0.05).

## Discussion

Mesenchymal stromal cells have become a widespread source of cells in regenerative medicine due to easy access to adipose tissue and their low immunogenicity and multidirectional differentiation potential. In the treatment study of ADSCs on porcine liver IRI injury, it was found that the ADSCs reduced the inflammatory response induced by I/R combined with partial hepatectomy in minipigs, improved liver function, promoted liver regeneration [23], and inhibited hepatocyte apoptosis and excessive autophagy [19, 22]. The results of this study showed that in vivo treatment of ADSCs as a graft could alleviate the excessive autophagy injury of hepatocytes caused by hepatic IRI combined with hepatectomy. The findings of this study are consistent with those of Ge et al. However, there were still many safety considerations for the transplantation of MSCs in the clinic, including immunocompatibility, tumorigenicity, and emboli formation [24, 25]. ADSCs secrete a variety of cytokines in paracrine or autocrine ways [26]. Hsiao S T et al. detected higher levels of insulin-like growth factor-1, vascular endothelial growth factor-D, and interleukin-8 in the ADSC-secretome compared with those in the secretome of other mesenchymal stromal cell populations, and the ADSC-secretome had more advantages in promoting angiogenesis [27]. Now, our study has demonstrated that the ADSC-secretome not only reduced the expression levels of alanine transaminase, aspartate transaminase, and alkaline phosphatase and had obvious therapeutic effects, but also attenuated inflammation and activated the STAT3 signaling pathway to promote liver regeneration in a model of hepatic I/R combined with partial hepatectomy [28]. In this study, we observed that the ADSC-secretome improved the liver damage in a porcine hepatic I/R and hepatectomy model by alleviating hepatocyte autophagy.

Autophagy is a highly conserved biological process that maintains cell homeostasis and viability by recycling and reusing energy. However, it also plays a special role in the cell death process [29]. In extreme cases, such as acute liver injury or IRI [30–32], excessive and long-term upregulation of autophagy leads to the destruction of essential cellular proteins and organelles, further leading to cell death [7]. In special contexts, autophagy or autophagy-relevant proteins may help to induce apoptosis or necrosis, and autophagy has been shown to excessively degrade the cytoplasm, leading to autophagic cell death [33]. Lee S C et al. found that the ADSC-secretome alleviated liver damage and improved the liver microenvironment after hepatic IRI in mice [34]. A study on hepatic IRI therapy showed that hepatic injury was alleviated by reducing the level of excessive autophagy and hepatocyte apoptosis caused by hepatic IRI [35]. Therefore, autophagy is closely related to the pathogenesis of hepatic IRI. With this in mind, we explored the regulatory effect of the ADSC-secretome on hepatocyte autophagy in hepatic IRI. In the autophagic response, Beclin-1 recruits PI3KC3 (Vps34) to form a protein complex [36, 37]. The formation of Beclin-1 complexes opens the production of autophagosome membranes. The execution of autophagy requires two ubiquitin-like conjugation systems: the ATG8 (LC3) conjugation system and the ATG12-ATG5 conjugation system. LC3 is the only mammalian protein known to bind stably to the autophagosome membrane. LC3I is cleaved by autophagy-related proteins to form the autophagosome marker molecule LC3II, which is present in autophagosomes and is one of the molecular markers of autophagy. The content of LC3II is proportional to the degree of autophagy. ATG7 activates ATG12 and binds to ATG5 under the action of ATG10 to form the ATG12-ATG5 complex. In ubiquitin-binding reactions, the ATG5-ATG12 complex promotes the formation of ATG8-PE in a manner similar to the function of E3 enzymes [38]. The multifunctional protein p62 (SQSTM1) plays an important role in signaling and autophagic degradation. Therefore, we examined the gene and protein expression of autophagy-related factors Beclin-1, LC3II/LC3I, ATG5, ATG12, and p62 to observe the changes in autophagic activity induced by hepatic I/R and hepatectomy. We observed an increase in the Beclin-1, ATG5, and ATG12 mRNA and the Beclin-1, LC3II/LC3I, and ATG5 protein levels, and a decrease in the p62 mRNA and protein levels in liver tissues after IRI, which were significantly alleviated by the ADSC-secretome. Combining the above results with the results of histopathology and TEM, we found that the level of hepatocyte autophagy increased and the hepatocyte injury was serious after hepatic IRI, while the hepatocyte injury was significantly alleviated, and the level of hepatocyte autophagy significantly decreased after ADSC-secretome intervention treatment. These findings provide a potential new strategy for improving hepatic IRI. Therefore, further experiments are needed to explore the mechanism underlying the ADSC-secretome therapeutic effects on hepatocyte autophagic injury induced by hepatic I/R.

The PI3K/Akt/mTOR signal transduction pathway is involved in the negative regulation of autophagy. [39, 40]. PI3K/Akt controls cellular functions, including mTOR, by regulating the expression of many downstream molecules. mTOR is a molecular target of rapamycin in mammalian cells and an important factor regulating cell growth and metabolism [41]. Activation of mTOR can inhibit autophagy. In particular, phosphorylated Akt activates mTOR, which negatively regulates autophagy by inhibiting the downstream molecule ULK1 complex. We found that the PI3K/Akt/mTOR pathway was inhibited after hepatic I/R and hepatectomy, while the gene expression levels of PI3K, Akt, mTOR and the protein expression levels of p-Akt and p-mTOR were significantly increased after the ADSC-secretome intervention. Therefore, combined with the above autophagy-related results, we conclude that the ADSC-secretome may inhibit the excessive autophagy of hepatocytes after hepatic I/R and hepatectomy by regulating the PI3K/Akt/mTOR pathway to alleviate the autophagic injury of hepatocytes.

Thus far, the ADSC-secretome has been found to have good therapeutic effects in many studies [42, 43]. As a substitute for ADSCs, the ADSC-secretome not only overcomes the limitations of cell therapy, but also maintains its advantages. Therefore, these cell-free products have the potential to replace cell transplantation therapies. The results of comparison of the ADSC-secretome and ADSCs in the regulation of hepatic IRI-induced excessive autophagy showed that after transplantation of the ADSC-secretome and ADSCs, the excessive autophagy in hepatocytes decreased, and there was no significant difference in autophagy levels between these two treatments. Both the ADSC-secretome and ADSCs had protective effects on hepatic autophagy-induced injury. Therefore, as a collection of cytokines secreted by ADSCs, the ADSC-secretome may play a major role in the therapeutic application of ADSCs to hepatic IRI-induced excessive autophagy.

This study has limitations. Our current study is a preliminary study on the effect of ADSC-secretome on excessive autophagy in hepatocytes caused by IRI combined with hepatectomy in pig livers. Therefore, only limited analysis and short-term observations were made. Our study demonstrated the significant efficacy of ADSC-secretome in hepatic IRI injury. As a cell-free therapy product with high safety and manufacturability, it is expected to replace ADSCs in future clinical applications. Therefore, further research on the protective mechanism of ADSC-secretome against hepatic IRI injury is of great significance for the clinical promotion of ADSC-secretome.

## Conclusion

This study demonstrated that the ADSC-secretome inhibited the excessive hepatocyte autophagy induced by hepatic IRI, possibly by upregulating the PI3K/Akt/mTOR signaling pathway, thereby alleviating the hepatocyte pathological damages and ultrastructural changes. In addition, there was no significant difference between the ADSC-secretome and ADSCs groups in the levels of hepatocyte autophagy, suggesting that the ADSC-secretome may play a major role in the therapeutic effect of ADSCs on hepatocyte autophagic injury induced by hepatic IRI. These findings provide a new theoretical basis for the application of cell-free therapy to clinical practice.

## Supplementary Information

**Additional file 1**. Supplementary materials.

## Data Availability

Please contact the corresponding author for data requests.

## Abbreviations

ADSCs: Adipose-derived mesenchymal stromal cells
ADSC-secretome: Adipose-derived mesenchymal stromal cell-secretome
IRI: Ischemia–reperfusion injury
I/R: Ischemia–reperfusion
