# Supplementary material for: Protective effect of adipose-derived stromal cell-secretome attenuate autophagy induced by liver ischemia–reperfusion and partial hepatectomy
Source: Stem Cell Res Ther. 2022 Aug 20;13:427. doi: 10.1186/s13287-022-03109-2 (PMC9392224; doi:10.1186/s13287-022-03109-2)
Supplement: Supplementary file 1 — Additional file 1. Supplementary materials. [file 13287_2022_3109_MOESM1_ESM.docx]

Additional File 1

Table A: ADSC-secretome total protein test results

| Variables | N1 | N2 | N3 | N4 | N5 |
| --- | --- | --- | --- | --- | --- |
| TP(mg/ml) | 0.32±0.05 | 0.34±0.05 | 0.34±0.03 | 0.33±0.03 | 0.31±0.02 |


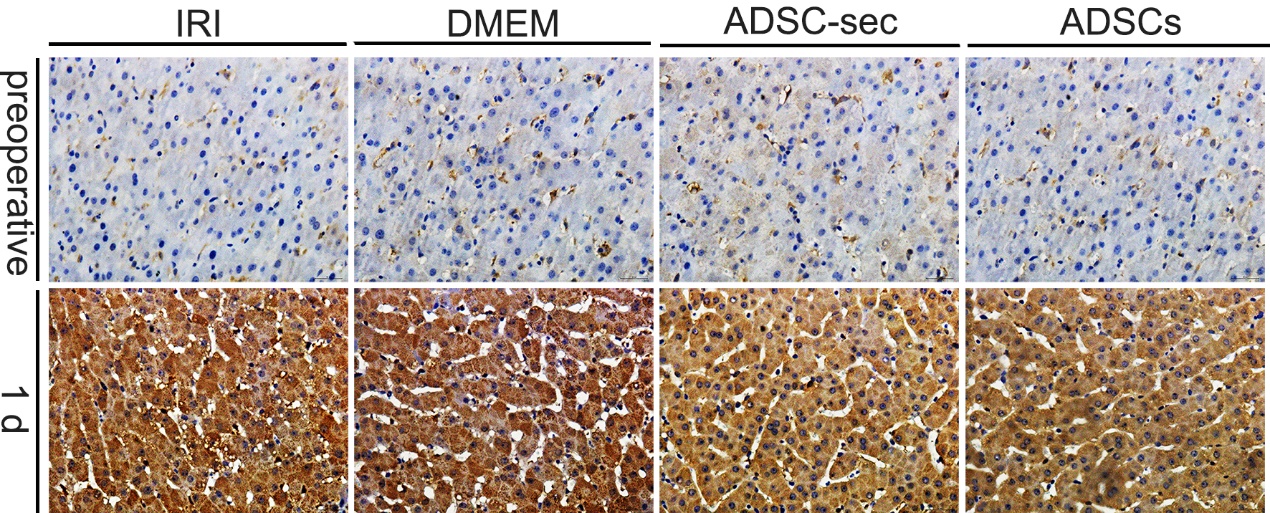


Figure A: Representative immunohistochemical images showing in situ LC3-Ⅱ expression . IRI: ischemia-reperfusion injury; ADSC-sec: adipose-derived mesenchymal stromal cell-secretome; ADSCs: adipose-derived mesenchymal stromal cells (Magnification × 400).
